# Supplementary figures and images for: Reticular adhesions are assembled at flat clathrin lattices and opposed by active integrin α5β1
Source: J Cell Biol. 2023 May 26;222(8):e202303107. doi: 10.1083/jcb.202303107 (PMC10225744; doi:10.1083/jcb.202303107)

Fig S1C

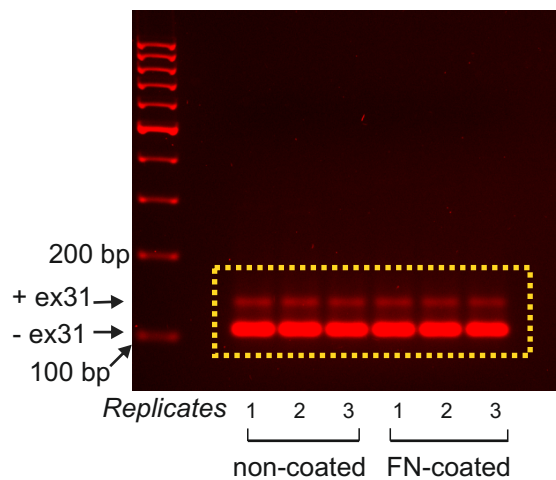

Supplement: SourceData FS1 — is the source file for Fig. S1. [file JCB_202303107_SourceDataFS1.pdf]

Fig S4C

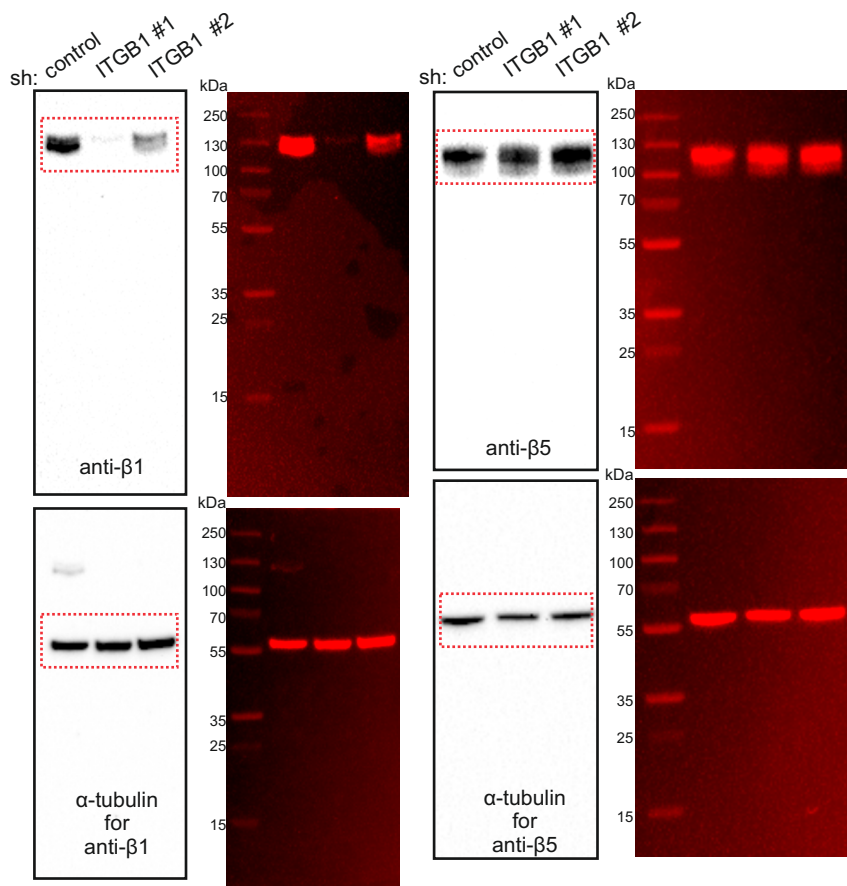

Supplement: SourceData FS4 — is the source file for Fig. S4. [file JCB_202303107_SourceDataFS4.pdf]

Fig S5C

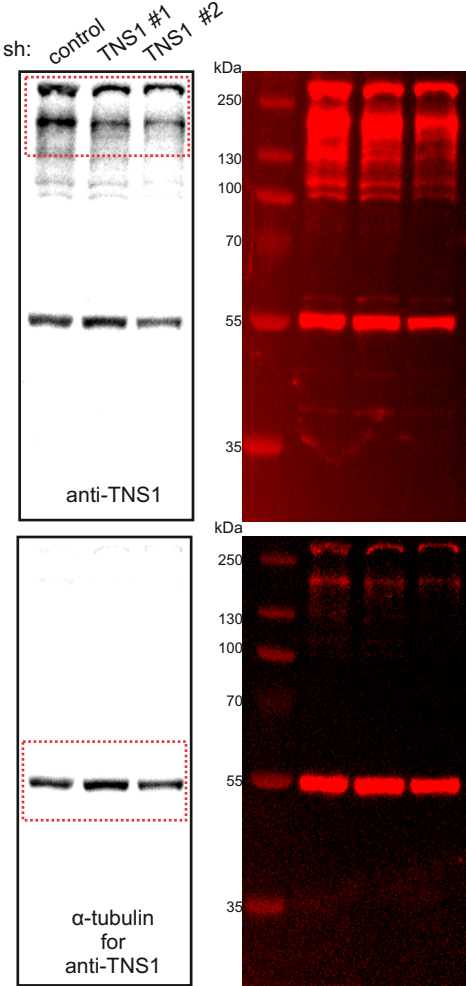

Fig S5F

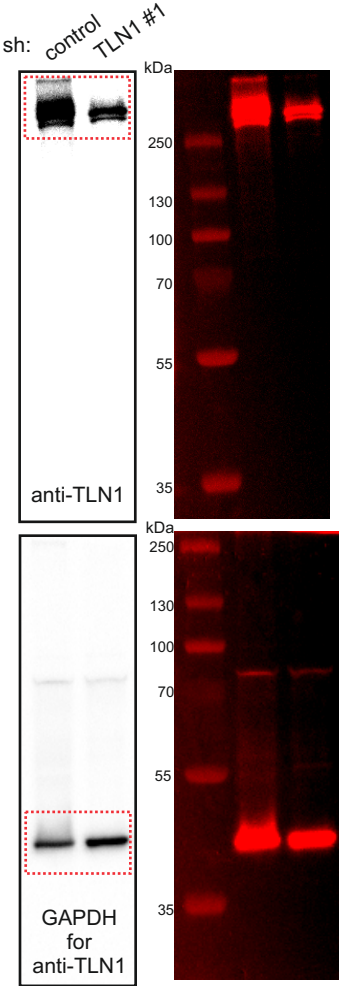

Supplement: SourceData FS5 — is the source file for Fig. S5. [file JCB_202303107_SourceDataFS5.pdf]
